# Increasing protocol suitability for clinical trials in sub-Saharan Africa: a mixed methods study

**DOI:** 10.1186/s41256-017-0031-1

**Published:** 2017-04-07

**Authors:** Nerina Vischer, Constanze Pfeiffer, Jennifer Kealy, Christian Burri

**Affiliations:** 1 Department of Medicines Research, Swiss Tropical and Public Health Institute, Socinstrasse 57, 4051 Basel, Switzerland; 2 University of Basel, Petersplatz 1, 4003 Basel, Switzerland; 3 Department of Epidemiology and Public Health, Swiss Tropical and Public Health Institute, Socinstrasse 57, 4051 Basel, Switzerland

## Abstract

**Background:**

The trial protocol is the most important document for clinical trials and describes not only the design and methodology of a study, but also all practical aspects. The suitability of the protocol has a direct impact on the execution and results of the trial. However, suitability is rarely addressed in trial practice and research. The aim of our study was to investigate protocol suitability and to identify suitability-enhancing measures for trials in sub-Saharan Africa.

**Methods:**

We used an exploratory mixed methods design. First, we interviewed 36 trial staff at different organisational levels in Ghana, Burkina Faso and Senegal. Second, we conducted an online survey among trial staff in sub-Saharan Africa to investigate trial protocol suitability based on the main themes distilled from the interviews.

**Results:**

Protocol suitability surfaced as a prominent topic in interviews with trial staff, critiqued for its lack of clarity, implementability and adaptation to trial participants as well as to the workforce and infrastructure available. Both qualitative and quantitative investigations identified local site staff involvement in protocol development as the most helpful mean of increasing protocol suitability. Careful assessment of the local context, capacity and cultures, and ensuring that staff understand the protocol were also cited as helpful measures.

**Conclusions:**

Our data suggests that protocol suitability can be increased by discussing and reviewing the protocol with trial staff in advance. Involving operationally experienced staff would be most useful. For multicentre trials, we suggest that at least one trial staff member from each of the sites with the highest expected recruitment rates be involved in developing the protocol. Carefully assessing the context prior to study start is indispensable to ensuring protocol suitability and should particularly focus on the workforce and infrastructure available, as well as the needs and availability of trial participants. To allow for protocol suitability enhancing measures, planners must allocate enough time for trial preparation and solicit feedback and information on context at an early stage. Such prospective planning would increase implementability, efficiency and quality of trials in the long run.

**Electronic supplementary material:**

The online version of this article (doi:10.1186/s41256-017-0031-1) contains supplementary material, which is available to authorized users.

## Background

Clinical trials are essential for developing new medicines and for improving disease management. From a public health perspective, clinical trials in sub-Saharan Africa (SSA), where high burdens of disease exist, are of particular importance. Trials conducted in this region face particular setting-specific challenges like deficits in infrastructure and skilled workforce, in addition to the already complex task of performing a trial. Specific additional challenges derive from the difficulties of getting patient information and consent [1] and the frequent involvement of children.

The most important document in a clinical trial is the trial protocol, the key document for planning, conducting, externally reviewing, overseeing and interpreting a study [2]. The trial protocol provides a rational for the trial, defines trial goals, processes and analysis methods and enables scientific and ethical review. A well-designed protocol is paramount for a successful clinical trial for several reasons. First, the study design described in the protocol significantly affects the costs of conducting the trial [3]. Second, protocol deficiencies may lead to amendments [2] and protocol deviations, which trigger queries and add to already heavy workloads. Protocol amendments are costly [4], may jeopardize data integrity [5] and trial participants’ safety, and cause delays and disruptions of the trial [4]. One study found that nearly half of all amendments may be avoidable [6]. Third, the length and complexity of protocols have increased dramatically over the past decades. Higher protocol complexity is directly associated with a greater number of amendments, lower levels of study performance [3, 7] and increases chances of non-adherence and, hence, of risk and low quality. The frequency of procedures per protocol has also increased at an annual rate of 8.7%, which adds to on-site work burdens [8]. The number of protocol deviations is one of the key measures for trial quality [9] and protocols are the most important instrument for quality risk management. In summary, the protocol largely determines quality, outcomes, efficiency and potential challenges in clinical trials. Getz et al. state that protocol design may hold the key to achieving higher levels of efficiency [8]. Despite the challenges mentioned above and the apparent importance of the protocol, there is little research on how to optimize the conduct of trials in the North as well as in resource-limited countries [10, 11]. Gheorghiade et al. criticise the limited data available to support best trial practices and that we only rely on experience and judgment [12].

To standardise the content and ensure the quality of trial protocols, the ICH E6 guideline “Good Clinical Practice” contains a full chapter on trial protocols [13]. The SPIRIT 2013 Statement (Standard Protocol Items: Recommendations for Interventional Trials) is a more comprehensive checklist of recommended items to include in a trial protocol [2]. This checklist was developed based on the argument that high-quality protocols facilitate proper conduct, reporting and external review of clinical trials, and that the completeness of trial protocols was often inadequate. In addition, the World Health Organisation’s website offers instructions for designing and formatting a research protocol [14]. TransCelerate Biopharma Inc. developed the freely available “Common Protocol Template” to improve consistency across the increasingly complex protocols [15]. Other free protocol templates are available on the web [16, 17]; selecting the correct template depends on local laws, regulations and the sponsor. All efforts described above mainly focus on the scientific part of the protocol, which is of most interest to researchers and reflects their training.

However, a trial protocol goes beyond describing the research design. It also serves as an operational manual and must satisfy experts from different backgrounds and disciplines [18]. To date, little emphasis has been placed on protocol operationalization. Getz and Campo state that protocol authors often transfer out-dated and unnecessary procedures into next study designs because they are routinely carried over from long-standing protocol templates and operating policies [7].

A key aspect of operationalization is protocol feasibility, which is customarily assessed after the protocol has been finalised by the sponsor. It is currently common practice in clinical trials to have a site feasibility assessment and/or a pre-study visit. During both visits, facilities are commonly assessed using a standard template in a checklist format that is often used across studies and is not tailored to the specific operational requirements of the trial protocol. On the global health trials webpage, such a protocol feasibility checklist is freely available [19]. To the best of our knowledge, there is only one study covering this topic [3]. This study highlights that protocol design feasibility is a topic of increasing interest to sponsor organizations and recommends more flexible and adaptive trial designs as well as more rigorous upfront planning and simulation.

In contrast to “feasible”, which is defined as achievable and possible, “suitable” is defined as fit for purpose [20]. Protocol suitability goes beyond feasibility and addresses not only technical aspects of the protocol but also considers settings, environments and culture, as well as effectiveness and efficiency of execution. These are of particular importance, as the protocol serves as a manual for health care providers [18]. Protocols that cannot be effectively executed may result in protocol deviations, amendments, quality issues and safety problems. While feasibility of trial sites is routinely assessed, protocol suitability is a new concept and rarely considered. Meeker-O’Connell et al. stress that improving protocol design, trial planning and quality oversight has a direct impact on inefficiencies like high costs and unsustainability [21]. With the rising complexity of trial protocols and the intense pressure on sponsors to accelerate development cycle times, suitability is becoming more important to alleviate execution burdens and ultimately improve trial conduct efficiency [8].

The study presented here covers protocol suitability for clinical trials in SSA that investigate poverty-related diseases. Ensuring protocol suitability is particularly difficult in these regions due to the geographical separation between sponsors and trial teams. To the best of our knowledge, this is the first study investigating trial protocol suitability in SSA. As clinical research is more established in South Africa and not exactly comparable with other SSA-countries, we excluded South Africa from our study [22].

Clinical trial staff in SSA implement the trial protocols in practice and can provide valuable insights regarding protocol suitability. Nevertheless, the experience of trial staff is rarely acknowledged in scientific publications. Furthermore, Cullati et al. stressed that more research on trial protocols using qualitative methods could shed light on the factors that facilitate the conduct of clinical research [23]. Hence, we assessed trial staffs’ perspectives by using an exploratory mixed methods approach, combining qualitative and quantitative methods. Mixing two methods has the capacity to strengthen results and conclusions [24]. The aim of our study was to identify how protocol suitability could be improved for clinical trials in SSA.

## Methods

### Study design

We used an exploratory mixed methods design, which is an ideal approach to exploring a topic for which no research has been carried out so far [24]. We started with a qualitative part, conducting key informant interviews with clinical trial staff working in SSA, to identify important variables of protocol suitability. In order to quantify identified variables, increase generalizability and explore correlations between variables, we followed up with a quantitative part comprising an online survey targeting trial staff. We used the connection approach, deriving major themes from the qualitative interviews and using them to develop and formulate the questions and answer options in the quantitative survey [25].

Ethical review exemption for the whole project was granted by the Ethics Committee of North-Western and Central Switzerland (EKNZ), based on the rationale that the research project did not involve access to or collect private, sensitive or health-related data or materials. For the qualitative study, we received full ethical clearance from the Ghana Health Service Ethical Review Committee (GHS-ERC: 18/09/14), the Comité d’Éthique sur la Recherche en Santé in Burkina Faso (N 2014-11-131) and the Comité National d’Éthique pour la Recherche en Santé in Senegal (n12/MSAS/DPRS/CNERS).

### Qualitative methods

We visited clinical research centres in Ghana, Burkina Faso and Senegal as they significantly contribute to public health activities in SSA and because the Swiss Tropical and Public Health Institute (Swiss TPH) has contacts with clinical research centres in these countries. In all three countries, we contacted the major clinical research centres that focus on poverty-related diseases and have a track record of completed clinical trials (no more than four such centres could be identified per country). In every country, we selected the first two research centres that agreed to our visit and ultimately conducted interviews in six centres, four of which were located in an urban setting and two in a rural setting. To ensure anonymity of interviewees, neither the names nor the exact locations of the clinical research centres are mentioned here. Interviews were open to all centre investigators, study coordinators, clinicians and quality assurance professionals with at least six months of experience in clinical research. In each centre, the sample was drawn with the assistance of a clinical researcher working in the centre, who approached eligible participants and acquainted them with this study.

Building on the literature and through pre-test with trial personnel working in SSA, we finalized the interview guide in an interdisciplinary team. Among other aspects, the guide consisted of the following questions:

- In your experience what is important for a good study protocol that is easy to implement?
- Could trial protocols be improved? If yes how and where?
- What is the influence of the study protocol on the trial?
- Who is writing the protocols you are working with?

All interviews were conducted by the first author of this paper. In Ghana, key informant interviews were conducted in English in December 2014. After translating the interview guide into French (including back-translation and terminology review), we conducted interviews in Burkina Faso and Senegal in March and April 2015. In each country, we considered having reached saturation of information in the number of interviews conducted when few or no new concepts were raised [26]. Unstructured observations, reflections during interviews and informal conversations with external monitors (who were on-site during our visit) were collected and documented in a field diary.

After explaining the purpose of the study and informing the participants of their right to withdraw from the study at any given time, participants gave written consent. Interviews were tape-recorded, transcribed verbatim and analysed using thematic analysis as per Braun and Clarke [27]. After repeated reading of the transcripts, initial coding was performed in MAXQDA 11. The analysis focused on the suitability of trial protocols. To ensure the analysis was reflective, notes were taken. We tested emerging themes from the data interrogation in further interview analyses. Themes were cross-tabulated to explore differences between countries and staff levels before finally defining and naming themes. This study adhered to consolidated criteria for reporting qualitative research (COREQ) [28].

### Quantitative methods

The survey was based on the key themes that emerged from the qualitative interviews, namely protocol characteristics, context adaptation and involvement of site staff. We developed the survey in a team that included clinical researchers, a statistician and a social scientist and discussed it with and received input from the European & Developing Countries Clinical Trials Partnership (EDCTP), a funder of investigator-initiated trials and active in SSA since 2003. The resulting survey (Additional files 1 and 2) consisted of single and multiple selection questions and ranking of table lists related to the following topics: protocol characteristics, adaptation of procedures and practical aspects in the protocol, measures to increase protocol suitability, and current and most helpful involvement in protocol development. The survey also captured the experience of participants and the degree to which measures were implemented. The survey was deployed using a web-based survey tool developed for researchers at the University of Basel (FlexiForm^©^).

The survey was piloted among 12 participants who had varying positions in the field of clinical research in SSA. As the relevance of the questions had already been tested in the qualitative interviews, the pilot run focused on the comprehensibility and clarity of questions.

In addition to covering the organisational levels reflected in the qualitative interviews, the survey also targeted pharmacists, lab coordinators and nurses working in clinical trials in order to consider a variety of perspectives and provide a bigger sample size. The English-language survey was translated into French, including back-translation and revision of terminologies. Invitations to participate were sent via email and contained the link to the English and French versions of the survey. Data collection took place from August 2015 until January 2016. A total of 294 survey requests were sent out by different organizations (Table 1) and all contained the appeal to forward the survey to team members.

**Table 1:** Survey distribution

Organisation distributing the survey	Number of trial staff in SSA receiving the survey by email
European and Developing Countries Clinical Trials Partnership (EDCTP)	80 investigators who had previously coordinated an EDCTP grant
Swiss Tropical and Public Health Institute	109 trial staff who worked on the RTS,S malaria vaccine trials in SSA 40 trial staff contacts from Swiss TPH
Two pharmaceutical companies	43 trial staff
European Federation of Pharmaceutical Industries and Associations (EFPIA)	22 trial staff

In the introductory text, we informed respondents that by filling in and pressing the “send” button they were giving consent to participate in the survey. In addition, respondents were assured of their anonymity and that it would not be possible to link the answers to their email-addresses. Respondents were informed that if they could not give a general answer, they should answer the question with reference to an on-going or most recent trial.

Categorical variables were described using absolute and relative frequencies and percentages. Explorative factor analysis (based on principal component analysis) with oblique rotation was performed on the survey items to identify potential associations between the protocol quality score (basing on different assessment variables) and personal characteristics of the respondents. The resulting factor scores were then regressed on personal characteristics of the respondents. Independent variables for the regression analyses were selected based on prior knowledge and experience; other potential covariates were screened but did not improve the model. A p-value smaller than 0.05 was considered statistically significant. Data were analysed using the statistical software STATA 14.

## Results

### Qualitative results

#### Participants

Thirty-six clinical trial staff participated in the key informant interviews (Table 2). Through open questions about efficiency, challenges and quality in the conduct of trials, protocol suitability emerged as a topic in the first five interviews in Ghana. To follow up on this topic, we added questions about protocol suitability to the remaining eight interviews in Ghana (no more than 13 trial staff were available for interviews in the two clinical research centres in Ghana). Qualitative research is centred on flexibility and the exploratory approach of the study enabled adjustments to follow up on an emerging topic [29]. We asked the questions on protocol suitability in clinical research centres in Burkina Faso and Senegal, as well.

**Table 2:** Role and experience of interviewees

		Ghana	Burkina Faso	Senegal
		(*n* = 8)	(*n* = 16)	(*n* = 12)
Role in trial	Investigators	2	8	6
	(*n* = 16)			
	Study coordinators	4	3	3
	(*n* = 10)			
	Clinicians	1	3	2
	(*n* = 6)			
	QA professionals	1	2	1
	(*n* = 4)			
Clinical research experience	0 to 1 year	0	1	1
	2 to 4 years	2	5	2
	5 to 7 years	1	0	1
	More than 7 years	5	10	8
Study Phase	Phase I (a or b)	2	10	2
	Phase II	2	13	3
	Phase III	6	13	6
	Phase IV	5	9	3
Type of trial	Drug trial	4	16	9
	Vaccine trial	7	13	7

#### Findings

##### Protocol characteristics

With high frequency, interviewees reported that a suitable trial protocol has to be clear to avoid methodological and procedural uncertainties that leave room for interpretation. Trial staff emphasized the importance of making protocols understandable for everyone on site, including less skilled staff like field workers, and consistent to avoid ambiguities and contradictions. A bit less frequently, interviewees mentioned the need to make protocols easy to implement, i.e. avoiding too many measurements at the same time. In addition, a few interviewees claimed that a logical flow was sometimes missing and called for well-structured protocols.

A few individuals preferred detailed protocols, citing an approved ability to understand and carry out procedures. Others favoured short protocols to facilitate work, without providing too many details that would only lead to amendments and non-adherence to protocol procedures. If protocols are too long, staff only read the section relevant to the work they have to execute.

*“A long document to read can cause a problem. Ideally, summarised protocols that get to the essential points could be better for both the researcher and the ethics committee. This facilitates understanding and implementation on the ground. So that's important.”*

— Study coordinator, male, Burkina Faso, centre two

Interviewees from French-speaking countries stated the need to translate the protocol into French, as technical staff are unlikely to understand English. According to interviewees, protocol translations were often of bad quality, leading to errors and ambiguities.

##### Importance of context adaptation

The importance of context adaptation came up in half of the interviews, independent of country and organisational level. One third of participants reported recently working with protocols that were not fully adapted to the setting.

*“I would tell you to try to really adapt to the realities of the countries. If you give us a typical European protocol that has to be reproduced here, I think we are going to have problems. We do not have the same manner of working. We do not have the same tools to work with. So it might be important to really see what is feasible in the country (…) If not, you will have many, many deviations afterwards, because we were not able to do that.”*

— Study coordinator, female, Burkina Faso, centre two

Interviewees gave various examples of missing context adaptation. First, their biggest concern was the needs of trial participants, which protocols sometimes failed to consider. Protocols should seek to burden trial participants as little as possible. For example, interviewees in all three countries asserted that trial participants felt uncomfortable with blood drawings; one Senegalese investigator said participants would rather accept four small tubes instead of one big tube hence, it is important to ensure that trials are as non-invasive as possible and to discuss limitations in advance. Another example referred to the heavy agricultural workload of local populations during the rainy season; many trials deliberately take place in this season due to high disease prevalence of malaria, for example. Thus, trial procedures should adapt to the time constraints of its participants. Second, interviewees found that socio-cultural norms and values were sometimes not respected in the protocol. Interviewees gave various examples of this, like asking trial participants about death or sexuality, which are taboo subjects in these settings. A few interviewees mentioned that trial participants would not answer these questions honestly. Other examples included performing HIV tests or pregnancy tests on minors or asking the name of neonates when neonates are not given names in their first seven days of life. One interviewee stressed the importance of having a male and a female area for clinical trials in Muslim environments. Interviewees believed that better adaptation to possibilities and attitudes of trial participants would also improve participants’ adherence to trial protocols and decrease losses to follow-up. Third, a few interviewees reported poor or no adaptation to local capacities, systems and/or the structure of the national health system. Staff experiences revealed that certain laboratory tests or the amount of workforce or expertise (e.g. presence of a psychologist) may not be available on site but were required by the protocol. A few interviewees claimed that the protocol timelines given for patient flow were written for ideal settings and circumstances, but not achievable in practice. Respondents were aware that full adaptation to the site was not possible for multicentre trials. However, they reported that for certain multicentre trials, they were allowed to adapt some sections or details to their setting, such as adapting the formulation of questions, which increased protocol suitability.

According to interviewees, protocols were not adapted to local realities because the ones who elaborated the protocol did not know the context. Hence, some procedures in the protocols were difficult to put into practice. Interviewee experiences revealed that it was best to adapt the protocol to the setting in the development phase, as it is far more challenging to adapt a finalized protocol.

##### Ideas for improving protocol suitability

Across countries and positions, interviewees’ strongest suggestion for increasing protocol suitability was to involve trial-site staff in the protocol development phase. This idea was raised by the majority of interviewees, often in an emotional manner.

“*So I think that involving the researcher in writing the protocol allows one to avoid challenges in the field. Because it is him [the researcher] who knows his setting well.”*

—Investigator, male, Burkina Faso, centre one

Interviewees had different suggestions on how best to involve the trial team in protocol development. A few participants proposed holding discussions with relevant stakeholders prior to writing the protocol, while others suggested writing the protocol together with the sponsor. A few recommended asking clinical trial staff to review the first draft of the protocol, with the aim of checking trial feasibility and providing added feedback. A few others preferred to wait until the protocol was finalized and then discuss the implementation of the trial in practice with the sponsor. For all trial staff, the objective of their involvement would be to ensure that the trial respected the realities of the setting and centre. Additionally, a few mentioned that their input regarding recruitment was of particular importance and would potentially accelerate recruitment rates.

At the time of the interviews, half of the interviewed trial staff was not involved in protocol development. Of the other half, most were involved only insofar as they received a draft protocol and corrected for coherency and applicability.

*“There have been protocol meetings on many studies, but it is not all the studies that you get the opportunity to be part of the protocol development and you find out that in instances when you are not part of which and where, you know a training did not trickle down well to the end-users, myself included, there may be errors caused.”*

— Study coordinator, male, Ghana, centre one

One Burkinabe investigator stated that participation in protocol development depended on the sponsor: if it was a pharmaceutical company, trial staff were not involved; if it was a university, the sponsor and the site staff developed the protocol together. A few Senegalese staff reported that only recently, they were asked to provide inputs before a protocol was finalized and submitted for ethical review. Most of the principle investigators (PIs) interviewed were allowed to give inputs during protocol development. One interviewee shared his opinion that these PIs should solicit input from the team.

*“One the PIs should have it [the protocol]. And the PI also has the responsibility of sub-delegate (…) if you were the PI, it doesn’t necessarily mean you are the technical person in some of the areas. So it is not enough for the PI to just look at it and say ‘oh the science is ok’, you need the technical people to look at it and then they advise ‘ok this way’.”*

— Quality assurance professional, male, Ghana centre one

The majority of trial staff agreed that not only the PI but also technical staff, like statisticians, data managers and trial nurses, should be involved in protocol development to prevent trial designs or data collection approaches which are difficult to implement. Others expressed the following sentiment:

*“When we work with a pharmaceutical company, it's difficult to get everyone involved. But at least the PI may be involved.”*

— Investigator, male, Burkina Faso, centre two

Trial staff cited additional benefits of their involvement to trial efficiency. According to interviewees, their involvement would decrease the number of amendments, help to find redundancies in the trial processes and improve the preparation of staff for the trial. They were dissatisfied with only executing protocols and claimed that collaboration was missing. Trial staff was also of the opinion that staff motivation would increase if they were allowed more influence on the protocol.

Finally, two interviewees mentioned that protocols should be written by investigators and sponsors together, so that the investigators could learn protocol writing skills.

*“When the monitors come for the training, we go through documents and we say when such things in our setting cannot be done like that. Then we have to go back again. This is what I have criticized sometimes. We must amend, go back, start again. Because if you amend, we have to resubmit and so on. Whereas, if maybe we could tolerate that for some studies, you can select the site first and the whole protocol development process is done together with the site. This will allow one to take into account many aspects and once we start the process, we won’t need to go backwards anymore.”*

— Quality assurance professional, male, Burkina Faso, centre two

Other ideas for increasing protocol suitability were also presented with some frequency. One such idea included conducting a test run with a dummy participant to identify and tackle difficulties in advance, coordinate activities and ensure that everyone knows their responsibility before recruitment starts.

*“Then we realized that the test run was our secret. That was our success because we had virtually identified all the possible problems, looked at how they could be resolved before the real test.”*

— Quality assurance professional, male, Ghana, centre two

A few interviewees suggested including trial participants’ perspectives in the protocol development, as patient challenges occur very frequently, e.g. during informed consent and follow-up. Involving trial participants in discussions and knowing their perspectives would help to increase protocol adherence, according to interviewees. Having “lessons learned” meetings after trial completion and providing sponsors with information about what went wrong was also deemed to have a positive influence on future trials. According to interviewees, identifying weak spots and finding solutions prior to writing the next protocol would avoid repeating the same mistakes and allow staff to profit from experience.

### Quantitative results

#### Participants

The final survey sample size was 110. Eleven records were excluded because these respondents indicated a country outside SSA as their main work place. Characteristics of the respondents are presented in Table 3. There were high proportions of PIs (26.4%) and trial staff with more than seven years of clinical research experience (49.1%). The majority of respondents worked in clinical research centres (71.8%) and 53.2% spent more than 75% of their working time on clinical trials. The distribution of survey participants across countries (Table 4) reflected the number of clinical trials conducted in different countries [30]. Only Malawi, Zimbabwe and Nigeria were underrepresented in our survey. We asked survey participants to forward the survey to colleagues working in clinical research, thus the total number of surveys distributed is unknown and we cannot calculate a response rate.

**Table 3:** Role and experience of survey participants

	Number of participants, *n* (%)
Recent primary role in clinical research	
Principle investigator	29 (26.4)
Sponsor-investigator	11 (10.0)
Investigator	16 (14.6)
Clinician	14 (12.7)
Quality assurance professional	7 (6.4)
Study coordinator	22 (20.0)
Pharmacist	3 (2.7)
Lab coordinator	7 (6.4)
Trial nurse	1 (0.9)
Institution	
Centre	79 (71.8)
Hospital	12 (10.9)
Field site	7 (6.4)
University	4 (3.6)
Other	6 (5.5)
Missing	2 (1.8)
Experience in clinical research	
0 to 1 year	4 (3.6)
2 to 4 years	20 (18.2)
5 to 7 years	32 (29.1)
More than 7 years	54 (49.1)
Experience in drug trials	91 (82.7)
Experience in vaccine trials	63 (57.3)
Sponsor	
Pharma companies	40 (36.4)
Other than pharma companies	29 (26.4)
Mixed	40 (36.4)
I do not know	1 (0.9)

**Table 4:** Distribution of survey participants per country

Country	Number of participants, *n* (%)
Kenya	23 (20.9)
Burkina Faso	18 (16.4)
Tanzania	13 (11.8)
Ghana	7 (6.4)
Uganda	7 (6.4)
Cameroun	6 (5.5)
Mali	6 (5.5)
Gabon	4 (3.6)
Mozambique	4 (3.6)
Gambia	3 (2.7)
Zimbabwe	3 (2.7)
Botswana	2 (1.8)
Ethiopia	2 (1.8)
Senegal	2 (1.8)
Benin	1 (0.9)
Congo	1 (0.9)
Zambia	1 (0.9)
Several countries in SSA	7 (6.4)
Total	110 (100)

#### Findings

##### Characteristics and context-adaptation of protocols

Protocol characteristics are presented in Table 5. Only one third (38.2%) of all respondents considered protocols as being easy to implement. About half of the survey participants rated trial protocols as being completely understandable, clear and consistent.

**Table 5:** Protocol characteristics

Protocol characteristics	not at all [%]	partially [%]	completely [%]	missing [%]	no opinion [%]
Understandable (for all staff levels)	3.6	43.6	51.8	0.9	-
Easy to implement	6.4	53.6	38.2	1.8	-
Clear (no uncertainties)	7.3	39.1	45.5	6.4	1.8
Well structured	1.8	22.7	71.8	2.7	0.9
Complex	15.5	50.9	29.1	2.7	1.8
Consistent (e.g. no ambiguities)	4.6	39.1	51.8	4.6	-
Well translated	6.4	21.8	40.9	10.9	20

Of the respondents, 65.1% considered the follow-up procedure described in the trial protocol to be well adapted to the setting, while 58.7% considered the inclusion and exclusion criteria to be well adapted (Table 6). Only about one third rated workforce availability (30.9%), daily clinical practice (32.1%) and available infrastructure (35.8%) as well adapted to the protocol requirements; thus, these elements were rated as having the lowest degree of context-adaptation. Some 13.6% considered participant incentives and 10% considered availability and needs of trial participants as marginally adapted to the context. By analysing the data from English and French-speaking countries individually we identified that trial staff in French-speaking countries rated the level of adaptation to the setting of the informed consent procedures, inclusion and exclusion criteria, incentives, medical procedures, safety reporting and follow-up as lower compared to staff from English-speaking countries. In turn, staff from English-speaking countries rated the level of adaptation to the setting of availability and needs of trial participants, daily clinical practice as well as workforce and infrastructure available as lower compared to staff from French-speaking countries (no details given). However, it has to be considered that only 25 trial staff from French-speaking countries participated in the survey.

**Table 6:** Adaptation of protocol procedures and in-protocol required resources to the setting

	marginally adapted [%]	partially adapted [%]	well adapted [%]	no opinion [%]
Informed consent procedure	3.6	39.1	56.4	0.9
Inclusion and exclusion criteria	3.7	36.7	58.7	0.9
Participant incentives for participation	13.6	45.5	36.4	4.6
Recruitment procedure	3.6	46.4	50.0	-
Data and information to be collected	4.6	36.7	57.8	0.9
Medical interventions	8.2	35.5	52.7	3.6
Medical procedures and decisions	7.3	39.5	51.4	1.8
Safety reporting and management	4.6	39.1	54.6	1.8
Follow-up procedure	2.8	30.3	65.1	1.8
Amount of workforce available	13.6	53.6	30.9	1.8
Infrastructure available	13.8	48.6	35.8	1.8
Availability and needs of trial participants	10.0	50.9	38.2	0.9
Daily clinical practice	8.3	56.9	32.1	2.8
Ethics committee system	8.2	46.4	44.6	0.9
Drug regulatory authority system	9.1	42.7	45.5	2.7

The majority of respondents (56%) mentioned that protocols were amended an average of three to five times per trial; 7.3% mentioned more than five protocol amendments per trial.

##### Measures to increase protocol suitability

**Fig. 1:**
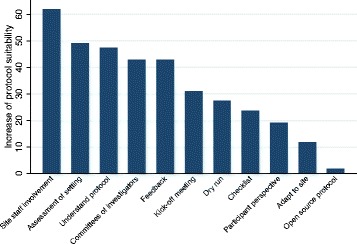
Measures to increase protocol suitability

When asked what measures would increase protocol suitability, involvement of local staff in the study planning and protocol development was rated as the most helpful option by respondents (61.8%) (Fig. 1).

This result was consistent across countries and positions. A related frequently selected approach to increase protocol suitability was to have committees of investigators in multicentre trials (42.7%), consisting of investigators from all participating sites. On average, respondents were more frequently working on multicentre trials than on single centre trials (77% spent at least 50% of their work time in multicentre trials). The second most helpful option mentioned was careful assessment of local context, capacity and culture by sponsors (49.1%). However, the adaptation of the protocol to site and health care specific systems in a single centre trial (11.8%) was only rarely selected. Overall almost half (47.3%) rated ensuring that everybody understands the protocol and knows his/her role and responsibility in the trial as one of the most helpful options, while the more concrete measures of having a kick-off meeting or a dry run were less frequently chosen (30.9 and 27.3%). Almost half (41.3%) reported to have had a kick-off meeting for all of their trials. Dry runs were less frequently implemented; 24.8% never had dry runs in any trial and 21.1% had dry runs in all trials. Soliciting feedback from the site on what went wrong in previous trials by sponsors was another popular option for respondents (42.7%) to increase protocol suitability. However, 40.4% had never had any “lessons learnt meetings”. Only 1.8% considered that the use of the open source protocol development technique would increase protocol suitability, but 73.4% had never heard of this technique.

Respondents were asked to tick the three options they considered most helpful for increasing protocol suitability. Because multiple selection was possible, 13.6% ticked more than three options. Percentages of answers from those that chose more than three options were compared with the answers of respondents who correctly filled in the survey; the ones that chose more than three options ranked checklist for practical steps, assessment of setting, inclusion of trial participants’ perspective and adaptation to site as slightly higher. However, including the ones that chose more than three options did not change the measures’ ranking order and, due to the small sample size, we included all answers in the final results and figures.

**Fig. 2:**
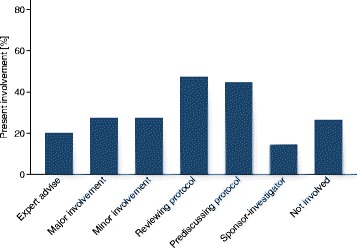
Present involvement of site staff

The qualitative interviews suggested that trial teams would consider their involvement in protocol development as highly important. Figure 2 shows that one quarter (26.4%) of trialists were not involved in protocol development at all; most were clinicians or study coordinators and only a few investigators and PIs. Reviewing the protocol was the most frequent manner of involvement (47.3%).

**Fig. 3:**
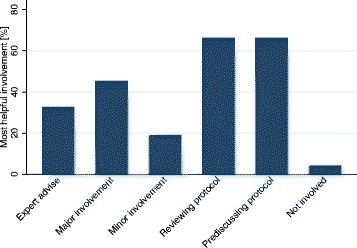
Most helpful involvement of site staff

Almost half (45.5%) indicated that it would be most helpful if they were heavily involved (major involvement) in protocol writing. However, as shown in Fig. 3; the majority considered reviewing protocols and participating in pre-discussions of protocols (both 66.4%) as optimal.

Factor analysis of the different assessment variables was performed to assess potential associations between protocol quality and personal characteristics. The third factor score was most strongly related to personal characteristics of the study participants and described the degree of implementability, understandability, clarity and structure of the trial protocol. The only statistically significant association found was the professional role of survey participants. Compared to other professional roles, the third factor score was on average significantly higher for PIs (difference = 0.43, 95%-confidence interval = 0.04 to 0.83, *p* = 0.033) and significantly lower for sponsor-investigators (difference = −1.10, 95%-confidence interval = −1.85 to −0.35, *p* = 0.005) and quality assurance professionals (difference = −1.22, 95%-confidence interval = −2.02 to −0.42, *p* = 0.003).

## Discussion

This study identified perceived deficits of protocol suitability and yielded several measures to enhance protocol suitability, as put forward by trial staff. According to trial staff, protocol suitability constraints included ambiguity, complexity and poor understandability for all staff levels. Staff mentioned lack of clarity in procedural descriptions and imprecise wording in protocols. Only one third of the survey respondents rated protocols as easy to implement. While context adaptation was a main theme in the qualitative interviews, survey respondents rated most trial aspects as rather well adapted, particularly inclusion and exclusion criteria and follow-up procedures. This finding surprised us and is inconsistent with the literature, which cites follow-up as a challenge [31–33]. The differences in the findings might be explained by the different methodologies used as literature findings base on authors’ personal reflections. In turn, respondents cited poor context adaptation of the protocol to the availability and needs of trial participants as a constraint. The importance of adapting projects to research participants’ cultural norms and values has been described elsewhere [1, 33, 34]. Staff perceived protocols as being too rigid for their settings. An example that was mentioned frequently in interviews was the importance of minimising blood draws. Trial participants in SSA are commonly scared of giving blood as blood is considered sacred, blood sampling is thought to make children ill and there are local rumours surrounding “blood stealing” and “blood selling” [1, 35, 36].

According to trial staff, visit windows and patient flow should also consider trial participants’ obligations, e.g. that harvesting takes place at the end of the rainy season. The lack of protocol adaptation to available site workforce and to daily clinical practice, of particular importance in hospital settings where trial activities are added to routine care, indicates that the work burden of trial implementation was underestimated by protocol developers.

The literature also stresses the importance of context adaptation for easy translation of protocol procedures into practice [37, 38]. Alsumidaie states that, “The sponsors design clinical trials expecting them to fit into study site operational infrastructures which leads to challenges like study procedures that are incompatible with how study sites operate” [39].

Context adaptation is more challenging in multicentre trials, as procedures must be uniform across sites to enable the required pooled data analysis. However, Thomason et al. state that standardisation of procedures across all sites within a trial in SSA is not always possible due to the differences in resources [33]. We argue that, for multicentre trials, the degree of adaptation to the context has to be considered carefully to avoid protocols that are overly site specific but that consider the cultural, social, economic and political differences between the sites involved. We suggest identifying commonalities among the sites involved and accounting for differing socio-cultural norms, but we acknowledge that this is difficult.

### Measures to improve protocol suitability

**Fig. 4:**
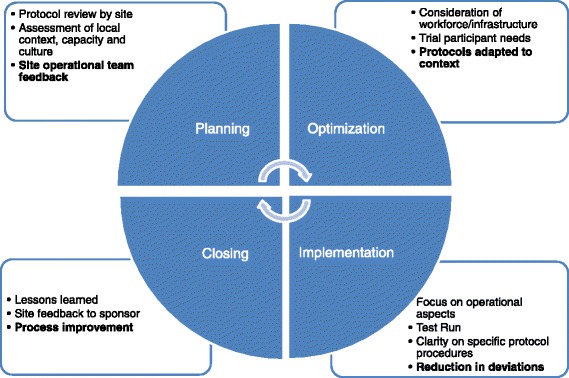
Implementation of measures to increase protocol suitability

Site staff identified a number of measures to improve the suitability of protocols, described below (Fig. 4).

#### Involvement of site staff in protocol development

In both qualitative and quantitative results, local trial staff involvement in developing the protocol came up and was rated as the most helpful option for increasing protocol suitability. The importance of site staff involvement in trial planning has also been stressed in the literature [11, 21, 32, 39]. Eastabrook et al. state, “Given the important role of site staff for overall trial success it is critical to understand their preferences and experiences” [40], while Alsumidaie promotes trial site involvement to create clinical trials that work operationally while reducing risks [39].

Half of the interviewees and three-quarters of survey respondents (half of survey respondents were investigators) had been previously involved in protocol development in some manner. It is important to carefully choose the composition of the trial team involved in this process. While key opinion leaders may give detailed scientific input, they are often not the ones carrying out the work on site. We agree with the respondents who suggested involving operationally experienced staff as particularly useful to increase suitability. Ideally, technical staff from various functional areas (investigator/clinician, study coordinator, pharmacist, lab coordinator and data manager) should be involved, though this might not be possible for all sites in a multicentre trial. A popular option to increase protocol suitability for multicentre trials, as revealed in the survey, was to form protocol writing committees consisting of one investigator from each site. Indeed, involving an operationally experienced investigator who solicits feedback from his/her team and communicates the outcome to the sponsor would lead to optimized protocols. Multicentre trials on poverty-related diseases do not usually involve an extensive number of trial sites, hence, it should be possible to solicit feedback from all sites in the planning phase of such trials. In cases where it is not possible to involve one investigator from each site, at least the sites expected to have a high enrolment rate should be involved in protocol development. Mbuagbaw et al. recommend selecting national coordinators to participate in the conception of multicentre trials [11]. The assistance of a social scientist would also help to inform protocol development by identifying context specificities. In line with this, Cooper et al. also suggest the use of qualitative research to identify the acceptability of the trial protocol among other things [41]. The meningitis vaccine project shares a positive experience of bringing trial teams together at meetings in the preparatory phase, which empowered the team and fostered communication between sites [32]. In addition, this approach enabled planners to anticipate and resolve operational issues and minimize the number of protocol deviations.

Trial staff rated reviewing and pre-discussing the protocol as the most helpful way to participate in protocol development. This is in line with Alsumidaie, who states that involvement is mainly about obtaining feedback on how to better operate the study [39]. As the sponsor is responsible for the research question and scientific aspects of the protocol, the trial team could provide valuable input in terms of protocol clarity, implementability and adaptation to trial participant needs. The latter is in line with literature stating that staff input would be particularly important for recruitment and follow-up of trial participants [32] as well as feasibility of scheduled study visits [42]. We consider it essential, that the site, in turn, is transparent and realistic in terms of their capacities.

In addition to increasing protocol suitability, trial staff mentioned several additional advantages of being involved in the protocol preparation process. First, developing an appropriate protocol is a discipline that requires training [31] and, according to interviewees and the literature, involving local trial staff in this process builds capacity and confidence [31, 43]. As there are only a small number of locally initiated trials [44] and limited career perspectives in clinical research [1, 45], this is a crucial skill for investigators to acquire. Second, staff saw being involved in protocol development as a way of improving their preparedness for the trial. Third, having an influence on the project would increase trial staff’s motivation, as opposed to having a project forced upon them. In contrast to a top-down approach, a participatory approach fosters ownership of the trial [38, 43] and is also recommended by the transboundary research principles of the Commission for Research Partnerships with Developing Countries (KFPE) [46]. Experiences from the meningitis vaccine project also show that working closely with study staff can be empowering, strengthen team spirit, boost staff motivation and increase everyone’s commitment [32]. Fourth, it has been stated that mobilising collective intelligence of various people for research protocols is a great benefit for research [47].

#### Assessment of the context and setting

More careful assessment of the local context, capacity, and culture by the sponsor was rated as the second most helpful measure to increase protocol suitability. Experiences from a trial in the Gambia are in line with this; the authors state that baseline situation assessments are required as each trial and site is unique [48]. For this purpose site assessments and pre-study-visits are commonly performed by sponsors or contract research organizations. While it is common practice to use standard templates (checklists), information collected this way is of limited value since they are frequently focusing on issues influencing trial feasibility. Instead, the questions should be tailored to the trial and the setting, i.e. to the suitability. This includes, for example, a more thorough assessment of the socio-cultural context, local laws and customs where the trial will be conducted and identification of risks and needs upfront. This is in line with current trends toward risk-based approaches, including risk assessments in clinical development [49].

Our data suggest improvement of both, feasibility and suitability assessments with a focus on workforce, infrastructure (e.g. equipment) available and on availability of trial participants, respectively. Some established clinical research centres in SSA have community advisory boards (CABs) for community engagement and to inform the community appropriately about the study [50]. In view of our findings, it might be helpful to involve the CAB at the conception stage of a trial, to allow for socio-cultural adaptation. Additionally, we found that engaging key staff from different organizational levels during the visits was beneficial. It is important to allocate enough time for such visits and ensure that they are conducted at an early project stage, where changes can still be incorporated. Moreover, it is important that the monitors performing these visits are well qualified, having both the requisite therapeutic knowledge and cultural sensitivity on top of the generally required knowledge of processes, protocols, regulations, laboratories and experience in clinical research.

The two measures “involvement of site staff in protocol development” and “assessment of context and setting” are not only increasing suitability but also feasibility. However, these measures go beyond feasibility through enabling effective and efficient execution of the trial and considering settings and cultures by following the protocol.

#### Good understanding of trial protocols

Making sure that everyone understands the protocol and knows his/her role and responsibility in the trial is an important factor, as put forward by trial staff and the literature [51]. However, it is challenging in practice, as often staff in SSA, particularly in trials on neglected diseases, have neither a medical background nor prior experience in clinical research [10]. A survey on site initiation visits confirmed our findings, as protocol specific training emerged as a main request by trial staff [40]. The initiation visit presents an ideal opportunity for the site staff to go through each trial procedure in detail and discuss the operationalization of the protocol with the monitor. To coach the team, ensure compliance with the trial protocol and help to correct practices, ideally, the monitor should remain on site during the first few days of recruitment or re-visit the site shortly after recruitment has started. Similarly, Tinto et al. suggested that the Good Clinical Practice trainer supports the trial team in resource-limited settings during trial start [52].

### Advantages of prospective planning

We are aware that the suggestions presented here to increase protocol suitability involve costs and might cause study start delays. However, a recent study by CTTI (Clinical Trials Transformation Initiative) confirmed that to overcome inefficiencies, an approach that emphasizes error prevention over remediation should be the norm [21]. Currently, the intense monitoring, auditing and inspecting processes test for errors during the trial rather than prospectively identifying, preventing and correcting them. In their quality by design project, the CTTI authors suggest, in line with our findings, that protocol issues should be identified early to minimize their impact and to describe the infrastructure, resources and training needs of the site [53]. Another study showed that to ensure data integrity, training and motivating sites is much more cost-effective compared to 100% source data verification [54]. To ensure quality, CTTI encourages critical thinking, addressing implementation challenges proactively and incorporating lessons learned into other trials as a means of continuous improvement. Protocol suitability-enhancing measures may also reduce the number of amendments, minimizing its negative impact on costs, duration, and quality of trials — particularly important given that the majority of survey respondents indicated three to five amendments, on average, per protocol.

Despite the intense pressure on sponsors to accelerate drug development [8], realistic timelines and sufficient time for trial preparation is important for implementing protocol suitability enhancing measures. This is in line with CTTI’s assertion that, “Rewarding trial teams who minimize the time to first patient enrolled may serve as a disincentive to devoting time to identifying and preventing errors that matter through trial design” [42].

To ensure that site staff involvement does not delay protocol development and that the process is as efficient as possible, it is important that one person leads the process on the sponsor side and that the protocol development process is clearly defined. The global health trials webpage offers a concept protocol template that provides a format for recording discussions and for presenting a protocol to stakeholders at an early stage [19]. Another promising tool for cost-effective and efficient involvement of stakeholders in protocol development is SWOG, a web-based protocol writing system with integrated support for collaborative reviewing and editing [55]. This tool enables sponsors and trial teams to see each other’s comments and reactions immediately, despite the geographical separation, which is particularly large for clinical trials in SSA [18]. The objective of the software developer was to increase the natural collaborative protocol writing process and facilitate interactions and communications among protocol writers [55].

### Internal validity

Due to time restrictions in this investigation, we covered the site teams’ perspective without incorporating the sponsors’ view. The interviewer of the qualitative interviews is a female Swiss scientist, which may have biased interviewees towards giving a positive answer. The survey sample is a convenience sample, as it is impossible to eliminate nonresponse bias in online surveys. However, triangulation of perspectives through mixed methods approach strengthened our findings. As EDCTP, pharmaceutical companies and Swiss TPH sent out the survey and are simultaneously potential sponsors or funders, survey respondents might have had a tendency to answer questions in a manner that would be viewed favourably by these organisations. However, we tried to mitigate this concern by ensuring anonymity in the survey’s introductory text. Another limitation was incomplete responses received for a few questions. Lastly, we acknowledge that according to the sample size calculation, 200 survey answers would have been required, but despite many efforts we only received 110 answers. Possible reasons were poor internet connectivity and time constraints of trial staff. However, one should consider that the total number of clinical research staff working in SSA is likewise relatively small and we believe that our results are representative for established clinical research centres in SSA.

### External validity

We speculate that many of our findings are also applicable for Northern settings. Due to the lack of literature on the topic in general and particularly in SSA, the majority of literature cited in this manuscript is based on the Northern setting and is mostly in line with our findings. An example that is equally true for Northern and Southern settings is the practice of sponsors designing clinical trials, expecting them to fit to the trial site [39]. Based on trial experiences in Northern settings, Farrell et al. recommend that differing clinical practices, working environments, and governance regulations should be taken into account [38] and CTTI recommends involving different levels of site staff to increase the quality of the trial [56].

To the best of our knowledge, this is the first study on trial protocol suitability for clinical trials in SSA. We encourage further research on trial protocols and their non-scientific parts, in particular. We promote the exploratory mixed methods methodology in the context where little is known about the research topic, as this approach allows new and important themes to emerge and provides the flexibility to adapt to these themes in subsequent steps.

## Conclusions

By applying an exploratory mixed methods approach, we identified a lack of clarity, implementability and adaptation to trial participants, workforce and infrastructure as the main constraints of protocol suitability. We found that site staff involvement in protocol development, careful assessment of local context, capacity and culture as well as ensuring that staff understands the protocol are the most helpful measures towards increasing protocol suitability, according to trial teams. Including technical aspects into such preparations and site involvement would simultaneously also enhance the feasibility of trials in the reviewed context. Considering and involving the site’s input at an early stage of protocol development was deemed the best way to increase involvement, as the majority of trial staff did not seek major involvement in protocol development. Our data suggests that the measures presented increase implementability, efficiency and quality of trials in the long run, although it might slightly prolong the protocol development phase. We consider such an approach as particularly useful for clinical trials in SSA, as the protocols are mostly developed by Northern sponsors who might not be familiar with the setting.

## Additional files

Additional file 1: Text S1. Survey-trial protocol, English. (PDF 359 kb)

Additional file 2: Text S2. Survey-trial protocol, French. (PDF 416 kb)

Additional file 3: Data S3. Data survey protocol. (CSV 18 kb)

Additional file 4: Table S4. Code book, survey protocol. (PDF 108 kb)

## Abbreviations

CAB: Community advisory board
CRO: Contract research organization
CTTI: Clinical Trials Transformation Initiative
EDCTP: European & Developing Countries Clinical Trials Partnership
PI: Principle investigator
SSA: Sub-Saharan Africa
Swiss TPH: Swiss Tropical and Public Health Institute

## References

[1] Idoko OT, Kochhar S, Agbenyega TE, Ogutu B, Ota MO (2013). Impact, challenges, and future projections of vaccine trials in Africa. Am J Trop Med Hyg.

[2] Chan A-W, Tetzlaff JM, Altman DG, Laupacis A, Gøtzsche PC, Krleža-Jerić K (2013). SPIRIT 2013 statement: defining standard protocol items for clinical trials. Ann Intern Med.

[3] Getz K (2014). Improving protocol design feasibility to drive drug development economics and performance. Int J Environ Res Public Health.

[4] Tufts Center for the Study of Drug Development. Impact Report: Amendments reduce number of patients, but at high cost, longer study times. 2016. Available from: http://csdd.tufts.edu/files/uploads/Summary-JanFebIR2016_.pdf. Accessed 24 June 2016.

[5] Chan A-W, Tetzlaff JM, Gøtzsche PC, Altman DG, Mann H, Berlin JA, et al. SPIRIT 2013 explanation and elaboration: guidance for protocols of clinical trials. BMJ. 2013;346: e7586. doi:10.1136/bmj.e7586. 10.1136/bmj.e7586 PMC3541470 23303884

[6] Getz K, Stergiopoulos S, Short M, Surgeon L, Krauss R, Pretorius S, et al. The Impact of Protocol Amendments on Clinical Trial Performance and Cost. Ther Innov Reg Sci. 2016. doi:10.1177/2168479016632271. 10.1177/2168479016632271 30227022

[7] Getz K, Campo R (2013). Drug development study designs have reached the danger zone. Expert Rev Clin Pharmacol.

[8] Getz KA, Wenger J, Campo RA, Seguine ES, Kaitin KI (2008). Assessing the impact of protocol design changes on clinical trial performance. Am J Ther.

[9] Alsumidaie M. Eli Lilly Unveils Innovative Study Design Platform. 2014. Available from: http://www.appliedclinicaltrialsonline.com/eli-lilly-unveils-innovative-study-design-platform. Accessed 24 June 2016.

[10] Geldenhuys H, Waggie Z, Jacks M, Geldenhuys M, Traut L, Tameris M (2012). Vaccine trials in the developing world: operational lessons learnt from a phase IV poliomyelitis vaccine trial in south Africa. Vaccine.

[11] Mbuagbaw L, Thabane L, Ongolo-Zogo P, Lang T (2011). The challenges and opportunities of conducting a clinical trial in a low resource setting: the case of the Cameroon mobile phone SMS (CAMPS) trial, an investigator initiated trial. Trials.

[12] Gheorghiade M, Vaduganathan M, Greene SJ, Mentz RJ, Adams KF, Anker SD (2012). Site selection in global clinical trials in patients hospitalized for heart failure: perceived problems and potential solutions. Heart Fail Rev.

[13] International Conference on Harmonisation of Technical Requirements for Registration of Pharmaceuticals for Human Use. Guideline for Good Clinical Practice E6. 1996. Available from: http://www.ich.org/fileadmin/Public_Web_Site/ICH_Products/Guidelines/Efficacy/E6/E6_R1_Guideline.pdf. Accessed 20 Oct 2015.

[14] World Health Association. Recommended format for a Research Protocol. Available from: http://www.who.int/rpc/research_ethics/format_rp/en/. Accessed 15 Jul 2016.

[15] TransCelerate Biopharma Inc. Common Protocol Template. Available from: http://www.transceleratebiopharmainc.com/assets/common-protocol-template/. Accessed 24 June 2016.

[16] NHS. Protocol guidance and template for use in a clinical trial of an investigational medicinal product (CTIMP). 2015. Available from: http://www.hra.nhs.uk/about-the-hra/consultations-calls/closed-consultations/protocol-guidance-template-use-clinical-trial-investigational-medicinal-product-ctimp-consultation-use/. Accessed 24 June 2016.

[17] FDA and NIH. Clinical Trial Protocol Template. 2016. Available from: http://osp.od.nih.gov/sites/default/files/Protocol_Template_05Feb2016_508.pdf. Accessed 24 June 2016.

[18] Gennari JH, Weng C, McDonald DW, Benedetti J, Green S (2004). An ethnographic study of collaborative clinical trial protocol writing. Stud Health Technol Inform.

[19] Global Health Trials. Templates. Available from: https://globalhealthtrials.tghn.org/resources/templates/. Accessed 24 June 2016.

[20] Wikidiff. Feasible vs Suitable - What’s the difference?. Available from: http://wikidiff.com/suitable/feasible. Accessed 19 Jul 2016.

[21] Meeker-O’Connell A, Glessner C, Behm M, Mulinde J, Roach N, Sweeney F (2016). Enhancing clinical evidence by proactively building quality into clinical trials. Clin Trials.

[22] Irikefe V, Vaidyanathan G, Nordling L, Twahirwa A, Nakkazi E, Monastersky R (2011). Science in Africa: the view from the front line. Nature.

[23] Cullati S, Courvoisier DS, Gayet-Ageron A, Haller G, Irion O, Agoritsas T (2016). Patient enrollment and logistical problems top the list of difficulties in clinical research: a cross-sectional survey. BMC Med Res Methodol.

[24] Creswell J, Plano CV (2011). Designing and conducting mixed methods research.

[25] Zhang W, Creswell J (2013). The use of “mixing: procedure of mixed methods in health services research. Med Care.

[26] Charmaz K (2006). Constructing grounded theory. A practical guide through qualitative analysis.

[27] Braun V, Clarke V (2006). Using thematic analysis in psychology. Qual Res Psychol.

[28] Tong A, Sainsbury P, Craig J (2007). Consolidated criteria for reporting qualitative research (COREQ): a 32-item checklist for interviews and focus groups. International J Qual Health Care.

[29] Silverman D (2013). Doing qualitative research.

[30] ClinicalTrial.gov. Map of All Studies on ClinicalTrials.gov. Available from: https://clinicaltrials.gov/ct2/search/map/click?map.x=428&map.y=284. Accessed 27 June 2016.

[31] Mwangoka G, Ogutu B, Msambichaka B, Mzee T, Salim N, Kafuruki S (2013). Experience and challenges from clinical trials with malaria vaccines in Africa. Malar J.

[32] Marchetti E, Mazarin-Diop V, Chaumont J, Martellet L, Makadi M-F, Viviani S (2012). Conducting vaccine clinical trials in sub-Saharan Africa: operational challenges and lessons learned from the meningitis vaccine project. Vaccine.

[33] Thomason MJ, Spyer MJ, Joffe N, Boles J, Burke A, Wilkes HC (2015). Monitoring randomized clinical trials in Africa; pragmatic approaches and experiences from HIV trials. Clin Investig.

[34] Kuepfer I, Burri C (2009). Reflections on clinical research in sub-Saharan Africa. Int J Parasitol.

[35] Peeters Grietens K, Ribera JM, Erhart A, Hoibak S, Ravinetto RM, Gryseels C (2014). Doctors and vampires in Sub-Saharan Africa: ethical challenges in clinical trial research. Am J Trop Med Hyg.

[36] Idoko OT, Owolabi OA, Odutola AA, Ogundare O, Worwui A, Saidu Y (2014). Lessons in participant retention in the course of a randomized controlled clinical trial. BMC Res Notes.

[37] Institute of Medicine (US) Forum on Drug Discovery, Development, and Translation. Transforming Clinical Research in the United States: Challenges and Opportunities: Workshop Summary. 2010. Available from: http://www.ncbi.nlm.nih.gov/books/NBK50892/. Accessed 6 April 2016. 21210556

[38] Farrell B, Kenyon S, Shakur H (2010). Managing clinical trials. Trials.

[39] Alsumidaie M. 2015 a Year in Review: The Year of Clinical Trial Innovation. 2015. Available from: http://www.appliedclinicaltrialsonline.com/good-risk-based-management-begins-site. Accessed 23 Aug 2016.

[40] Jennifer Eastabrook DM, Shawn Patterson Baker, Ruth Cannata. Enhanced Site Training, Resources, and Communication. 2016. Available from: http://www.appliedclinicaltrialsonline.com/enhanced-site-training-resources-and-communication-during-clinical-trials-site-staff-perspectives. Accessed 28 June 2016.

[41] Cooper C, O’Cathain A, Hind D, Adamson J, Lawton J, Baird W (2014). Conducting qualitative research within clinical trials units: avoiding potential pitfalls. Contemp Clin Trials.

[42] Clinical Trials Transformation Initiative Quality by Design Project. CTTI Recommendations: Qualtiy by Design. 2015. Available from: https://www.ctticlinicaltrials.org/files/ctti_quality_by_design_recommendations_final_1jun15_1.pdf. Accessed 27 June 2016.

[43] Devasenapathy N, Singh K, Prabhakaran D (2009). Conduct of clinical trials in developing countries: a perspective. Curr Opin Cardiol.

[44] Franzen SR, Chandler C, Enquselassie F, Siribaddana S, Atashili J, Angus B (2013). Understanding the investigators: a qualitative study investigating the barriers and enablers to the implementation of local investigator-initiated clinical trials in Ethiopia. BMJ Open.

[45] Ndebele P, Blanchard-Horan C, Shahkolahi A, Sanne I (2014). Regulatory challenges associated with conducting multicountry clinical trials in resource-limited settings. J Acquir Immune Defic Syndr.

[46] Commission for Research Partnerships with Developing Countries (KFPE). KFPE’s Guide for Transboundary Research Partnerships. 2014. Available from: http://www.naturalsciences.ch/organisations/kfpe/11_principles_7_questions?_ga=1.249050371.561251459.1440766959. Accessed 22 Aug 2016.

[47] Methods in Research on Research (MiRoR). Individual Research Projects. 2015. Available from: http://miror-ejd.eu/individual-research-projects/. Accessed 28 June 2016.

[48] Cutts FT, Enwere G, Zaman SM, Yallop FG (2006). Operational challenges in large clinical trials: examples and lessons learned from the gambia pneumococcal vaccine trial. PloS Clin Trials.

[49] Brosteanu O, Houben P, Ihrig K, Ohmann C, Paulus U, Pfistner B (2009). Risk analysis and risk adapted on-site monitoring in noncommercial clinical trials. Clin Trials.

[50] Nyika A, Chilengi R, Ishengoma D, Mtenga S, Thera MA, Sissoko MS (2010). Engaging diverse communities participating in clinical trials: case examples from across Africa. Malar J.

[51] Lang T, Siribaddana S (2012). Clinical trials have gone global: is this a good thing?. PLoS Med.

[52] Tinto H, Noor RA, Wanga CL, Valea I, Mbaye MN, D’Alessandro U (2013). Good clinical practice in resource-limited settings: translating theory into practice. Am J Trop Med Hyg.

[53] Clinical Trials Transformation Initiative. Crititical to quality factors. 2015. Available from: https://www.ctti-clinicaltrials.org/toolkit/qbd/introduce-qbd/qbd-principles/explore-ctq-factors. Accessed 27 June 2016.

[54] Goldfarb N. Are site monitoring and data cleaning a waste of time? Journal of Clinical Research Best Practices. 2006;2(11):1–9.

[55] Weng C, Gennari JH, McDonald DW (2004). A collaborative clinical trial protocol writing system. Stud Health Technol Inform.

[56] Smith SK, Selig W, Harker M, Roberts JN, Hesterlee S, Leventhal D (2015). Patient engagement practices in clinical research among patient groups, industry, and academia in the united states: a survey. PLoS One.

