# Supplementary material for: Increasing protocol suitability for clinical trials in sub-Saharan Africa: a mixed methods study
Source: Glob Health Res Policy. 2017 Apr 7;2:11. doi: 10.1186/s41256-017-0031-1 (PMC5683382; doi:10.1186/s41256-017-0031-1)
Supplement: Supplementary file 1 — Text S1. Survey-trial protocol, English. (PDF 359 kb) [file 41256_2017_31_MOESM1_ESM.pdf]

Survey about clinical trial protocols

1. What was your most recent primary role in clinical research?

- Sponsor-Investigator
- Principle Investigator
- Investigator
- Clinician
- Position in quality assurance
- Study coordinator
- Pharmacist
- Lab coordinator
- Clinical trial nurse

2. For how many years have you been working in clinical trials?

- 0-1 year
- 2 - 4 years
- 5 - 7 years
- more than 7 years

3. In which diseases area are you working? (Please choose all that apply)

- [ ] Malaria
- [ ] Tuberculosis
- [ ] HIV
- [ ] Other neglected tropical diseases
- [ ] Non communicable diseases
- [ ] Other: - enter here -

4. In which kind of clinical trials are you involved in? (Please choose all that apply)

- [ ] Vaccine trials
- [ ] Drug trials
- [ ] Other: - enter here -

5. The protocols you have worked with are

	not at all	partially	completely	no opinion
understandable (for all staff levels involved)	○	○	○	○
easy to implement	○	○	○	○
clear (no uncertainties)	○	○	○	○
well structured	○	○	○	○
complex	○	○	○	○

consistent (e.g. no ambigueties or contradictions)	○	○	○	○
well translated (only for not english-speaking countries)	○	○	○	○

6. The protocols you have worked with are

	not at all	more or less	sufficiently	too much	no opinion
detailed	○	○	○	○	○
long	○	○	○	○	○

7. Optional field to comment on previous two questions:

8. How many amendements do you have in average per protocol?

- ○ 0
- ○ 1 - 2
- ○ 3 - 5
- ○ > 5
- ○ I do not know
- ○ Other: - enter here -

9. How well are the study procedures described in the protocol adapted to your specific setting? (1=poorly adapted, 5=well adapted)

	1	2	3	4	5	no opinior
Informed consent procedure including documentation	○	○	○	○	○	○
Inclusion and exclusion criteria	○	○	○	○	○	○
Participants incentives to participate in the trial	○	○	○	○	○	○
Recruitment procedure	○	○	○	○	○	○
Data and information to be collected	○	○	○	○	○	○
Medical interventions (e.g. ECG)	○	○	○	○	○	○
Medical procedures and decisions (e.g. administration of drugs, treatment of concomitant diseases and emergencies)	○	○	○	○	○	○
Safety reporting and management	○	○	○	○	○	○
Follow-up procedure	○	○	○	○	○	○

10. How well are the protocols adapted to...? (1=poorly adapted, 5=well adapted)

	1	2	3	4	5	no opinior
Amount of workforce available	○	○	○	○	○	○
Infrastructure available	○	○	○	○	○	○
Availability and needs of trial participants	○	○	○	○	○	○
Daily clinical practice	○	○	○	○	○	○
Ethics Committe system	○	○	○	○	○	○
Drug Regulatory Authority system	○	○	○	○	○	○

11. Optional field to comment on previous two questions:

12. Are you involved in the study planning of the clinical trials you are working in? (Please choose all that apply)

- Stimulating the topic as an expert
- Major involvement in protocol writing
- Minor involvement in protocol writing
- Reviewing the protocol
- Participating in prediscussion of protocol
- As a sponsor-investigator
- Not involved
- Other: - enter here -

13. In which role would your involvement be most helpful within the study planning of the clinical trials you are working in? (Please choose all that apply)

- Stimulating the topic as an expert
- Major involvement in protocol writing
- Minor involvement in protocol writing
- Reviewing the protocol
- Participating in prediscussion of protocol
- Not involved
- Other: - enter here -

14. Have you ever heard about open source protocol development?

- Yes, I have heard about it
- Yes, I have heard about it and was participating in an open source protocol development
- No, I have never heard of it
- Other: - enter here -

15. Please tick the top three options you think help or would help to increase the suitability of trial protocols? (Please tick three options)

- Sponsor to solicit feedback from site on what went wrong in previous trials
- More careful assessment of local context, capacity and culture by sponsor
- Include participant perspective in study planning
- Involvement of local staff in the study planning/ protocol development
- Use open source protocol developement technique
- Single center trials: Adapt the protocol to site and health care specific systems
- Multi center trials: Having committees which consist of investigators from all involved research centres
- Making sure that everybody understands the protocol and knows his role and responsibility in the trial
- Having a kick-off meeting before the study start where issues can be discussed and detected
- Having a dry run before the enrolment of the first patient
- Having a checklist for all the practical steps of the trial

16. Optional field to comment on previous question:

17. In which country do you work most of the time?

18. In what kind of institution are you working in?

- Clinical research centre
- Hospital
- Field site
- Other: - enter here -

19. What percentage of your working time is spent for work on clinical trials?

- 0 - 25%
- 26 - 50%
- 51 - 75%
- 76 - 100%
- Other: - enter here -

20. For which percentage of clinical trials have you had a dry run (definition: a practice of the trial activities with dummy participants before the enrolment of the first participant)?

- 0 %
- 25 %
- 50 %
- 75 %
- 100 %
- Other: - enter here -

21. For which percentage of clinical trials have you had a kick off meeting where issues were detected and discussec before the start of the study?

- 0 %
- 25 %
- 50 %
- 75 %
- 100 %
- Other: - enter here -

22. For which percentage of clinical trials have you had a lessons learnt meeting after the trial has ended?

- 0 %
- 25 %
- 50 %
- 75 %
- 100 %

- Other: - enter here -

23. Who was the sponsor of your study? ⓘ

- Mostly pharmaceutical companies
- Mostly other than pharmaceutical companies
- Mixed
- I do not know
- Other: - enter here -

24. What percentage of your trials are multicenter trials? ⓘ

- 0 %
- 25 %
- 50 %
- 100 %
- Other: - enter here -

Please help us prevent spam by entering the characters shown in the image below!

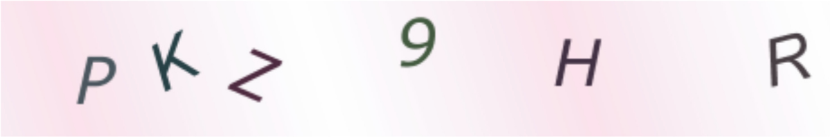

Send Reset
