# Supplementary material for: Increasing protocol suitability for clinical trials in sub-Saharan Africa: a mixed methods study
Source: Glob Health Res Policy. 2017 Apr 7;2:11. doi: 10.1186/s41256-017-0031-1 (PMC5683382; doi:10.1186/s41256-017-0031-1)
Supplement: Supplementary file 2 — Text S2. Survey-trial protocol, French. (PDF 416 kb) [file 41256_2017_31_MOESM2_ESM.pdf]

Enquête sur les protocoles d'essai clinique

1. Quel a été votre rôle principal le plus récent en recherche clinique?

- Promoteur-investigateur
- Investigateur principal
- Investigateur
- Clinicien
- Position en assurance qualité
- Coordinateur de l'étude
- Pharmacien
- Coordinateur de labo
- Infirmier d'un essai clinique

2. Depuis combien de temps travaillez-vous dans les essais cliniques?

- 0 - 1 an
- 2 - 4 ans
- 5 - 7 ans
- plus de 7 ans

3. Dans quelle aire thérapeutique travaillez-vous? (Plusieurs réponses possibles)

- Paludisme
- Tuberculose
- HIV
- Autres maladies tropicales négligées
- Maladies non transmissibles
- Autres (précisez): - saisir ici -

4. Dans quel type d'essais cliniques êtes-vous impliqué? (Plusieurs réponses possibles)

- Essais de vaccins
- Essais de médicaments
- Autres (précisez): - saisir ici -

5. Les protocoles sur lesquels vous avez travaillés sont

	pas du tout	en partie	complètement	pas d'opinior
compréhensibles (pour tous les niveaux de personnel impliqués)				
faciles à implémenter				
clairs (aucune incertitude)				
bien structurés				
complexes				

cohérents (pas d'ambigüités ou contradictions)	○	○	○	○
bien traduits (seulement pour les pays non-anglophones)	○	○	○	○

6. Les protocoles sur lesquels vous avez travaillés sont

	pas du tout	plus ou moins	suffisamment	trop	pas d'opinion
détaillés	○	○	○	○	○
longs	○	○	○	○	○

7. Si nécessaire, ajoutez vos commentaires sur les deux questions précédents:

8. Combien d'amendements avez-vous eu par protocole (en moyenne)?

- ○ 0
- ○ 1 - 2
- ○ 3 - 5
- ○ > 5
- ○ Je ne sais pas
- ○ Autres (précisez): - saisir ici -

9. Selon vous, les procédures d'étude suivantes décrites dans le protocole sont-elles adaptées à votre contexte spécifique? (1 = mal adaptées, 5 = bien adaptées)

	1	2	3	4	5	sans opinion
Procédure de consentement éclairé y compris la documentation	○	○	○	○	○	○
Critères d'inclusion et d'exclusion	○	○	○	○	○	○
Incitation des personnes à participer à l'essai	○	○	○	○	○	○
Procédure de recrutement	○	○	○	○	○	○
Données et informations à recueillir	○	○	○	○	○	○
Interventions médicales (par exemple ECG)	○	○	○	○	○	○
Procédures médicales et décisions (par exemple, administration de médicaments, traitement des maladies concomitantes et urgences)	○	○	○	○	○	○
Rapports et gestion de la sécurité	○	○	○	○	○	○
Procédure de suivi	○	○	○	○	○	○

10. Les protocoles sont-ils adaptés ...? (1 = mal adaptés, 5 = bien adaptés)

	1	2	3	4	5	sans opinic
à la disponibilité du personnel	○	○	○	○	○	○
à la disponibilité des infrastructures	○	○	○	○	○	○
à la disponibilité et aux besoins des participants	○	○	○	○	○	○
à la pratique clinique quotidienne	○	○	○	○	○	○
aux procédures des comités d`éthique	○	○	○	○	○	○
aux procédures des autorités de réglementation pharmaceutique	○	○	○	○	○	○

11. Si nécessaire, ajoutez vos commentaires sur les deux questions précédents:

12. A quel niveau êtes-vous impliqué dans la planification de l'étude des essais cliniques pour lesquels vous travaillez? (Plusieurs réponses possibles)

- Initiation du sujet en tant qu'expert
- Implication majeure dans l'écriture de protocole
- Participation mineure dans l'écriture de protocole
- Révision du protocole
- Participation aux pré-discussions sur le protocole
- En tant que promoteur-investigateur
- Pas impliqué
- Autres: - saisir ici -

13. Dans quel rôle votre implication serait-elle très utile au sein de la planification de l'étude des essais cliniques pour lesquels vous travaillez? (Plusieurs réponses possibles)

- Initiation du sujet en tant qu'expert
- Implication majeure dans l'écriture de protocole
- Participation mineure dans l'écriture de protocole
- Révision du protocole
- Participation aux pré-discussions sur le protocole
- Pas impliqué
- Autres (précisez): - saisir ici -

14. Avez-vous déjà entendu parler de l'élaboration de protocole en open source?

- Oui, j'en ai entendu parler
- Oui, j'en ai entendu parler et j'ai participé à un développement protocole en open source
- Non, je n'en ai jamais entendu parler
- Autres (précisez): - saisir ici -

15. S'il vous plaît, cochez les trois meilleures options qui, selon vous, aident ou aideraient à augmenter la facilité de la mise en œuvre des protocoles d'essais? (3 champs obligatoires)

- Que le promoteur sollicite des commentaires du site sur ce qui s'est mal passé lors des essais précédents
- Évaluation plus minutieuse de la culture, des capacités et des contextes locaux par le promoteur
- Inclure les perspectives du participant dans la planification de l'étude
- Implication du personnel local dans la planification de l'étude / le développement de protocoles
- Utiliser des techniques de développement de protocole en open source
- Pour les essais unicentriques: adapter le protocole aux procédures du site et au système de soin
- Pour les essais multicentriques: avoir des comités qui se composent d'investigateurs de tous les centres de recherche impliqués
- Faire en sorte que tout le monde comprenne le protocole et connaisse son rôle et sa responsabilité dans l`essai
- Avoir une réunion de lancement avant l'étude commence où les problèmes peuvent être détectés et discutés
- Faire un entrainement aux activités de l'essai clinique avec des participants factices avant l'inscription du premier participant

- [ ] Avoir une liste de contrôle pour toutes les étapes pratiques de l'essai

16. Si nécessaire, ajoutez vos commentaires sur la question précédente:

17. Dans quel pays travaillez-vous la plupart du temps?

18. Dans quel genre d'établissement travaillez-vous?

- Centre de recherche clinique
- Hôpital
- Site d'étude
- Autres (précisez): - saisir ici -

19. Quel pourcentage de votre temps de travail est consacré au travail pour les essais cliniques?

- 0 - 25%
- 26 - 50%
- 51 - 75%
- 76 - 100%
- Other: - saisir ici -

20. Pour quel pourcentage des essais cliniques auxquels vous avez participez, avez-vous fait un entrainement aux activités de l'essai clinique avec des participants factices avant l'inscription du premier participant?

- 0 %
- 25 %
- 50 %
- 75 %
- 100 %
- Other: - saisir ici -

21. Pour quel pourcentage des essais cliniques auxquels vous avez participez, avez-vous eu une réunion de lancement de l'étude où les problèmes ont été détectés et discutés avant le début de l'étude?

- 0 %
- 25 %
- 50 %
- 75 %
- 100 %
- Other: - saisir ici -

22. Pour quel pourcentage des essais cliniques auxquels vous avez participez avez-vous eu une réunion sur les leçons tirées après la fin de l'essai?

- 0 %
- 25 %
- 50 %

- 75 %
- 100 %
- Other: - saisir ici -

23. Qui était le promoteur de votre étude?

- Principalement des entreprises pharmaceutiques
- Principalement autre que les entreprises pharmaceutiques
- Mélangé
- Je ne sais pas
- Autres: - saisir ici -

24. Quel pourcentage de vos essais était des essais multicentriques?

- 0 %
- 25 %
- 50 %
- 100 %
- Other: - saisir ici -

Pour nous épargner des messages qui aboutissent dans les envois indésirables (spams), nous vous prions d'écrire le texte qui figure dans l'encadré ci-dessous.

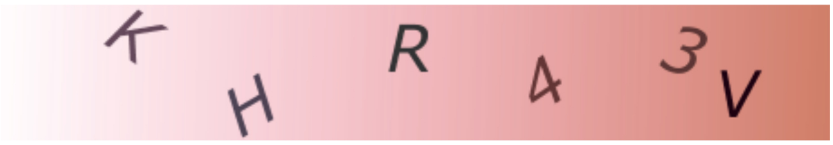

Envoyer Réinitialiser

Universitaet Basel Disclaimer - © 2004-2016 IT-Services > WAMS > WApp Created with *Flexi*Form 2
