# Supplementary material for: Increasing protocol suitability for clinical trials in sub-Saharan Africa: a mixed methods study
Source: Glob Health Res Policy. 2017 Apr 7;2:11. doi: 10.1186/s41256-017-0031-1 (PMC5683382; doi:10.1186/s41256-017-0031-1)
Supplement: Supplementary file 4 — Code book, survey protocol. (PDF 108 kb) [file 41256_2017_31_MOESM4_ESM.pdf]

Field name	Field label	Field values	Question number in survey
language	Filled in French or English	1 = French<br>0 = English	-
role	What was your most recent primary role in clinical research?	1=Sponsor-Investigator<br>2=Principle Investigator<br>3=Investigator<br>4=Clinician<br>5=Position in quality assurance<br>6=Study coordinator<br>7=Pharmacist<br>8=Lab coordinator<br>9=Clinical trial nurse	1
years	For how many years have you been working in clinical trials?	1=0-1 year<br>2=2 - 4 years<br>3=5 - 7 years<br>4=more than 7 years	2
aread	In which diseases area are you working? (Please choose all that apply)	1=Malaria<br>2=Tuberculosis<br>3=HIV<br>4=Other neglected tropical diseases<br>5=Non communicable diseases<br>6=Other	3
kind	In which kind of clinical trials are you involved in? (Please choose all that apply)	1=Vaccine trials<br>2=Drug trials<br>3=Other	4
underst	The protocols you have worked with are : understandable (for all staff levels involved)	1=not at all<br>2=partially<br>3=completely<br>4=no opinion	5
implement	The protocols you have worked with are : easy to implement	1=not at all<br>2=partially<br>3=completely<br>4=no opinion	5
cleare	The protocols you have worked with are : clear (no uncertainties)	1=not at all<br>2=partially<br>3=completely<br>4=no opinion	5
structur	Well structured	1=not at all<br>2=partially<br>3=completely<br>4=no opinion	5
complex	The protocols you have worked with are : complex	1=not at all<br>2=partially<br>3=completely<br>4=no opinion	5
consistent	The protocols you have worked with are : consistent (e.g. no ambigueties or contradictions)	1=not at all<br>2=partially<br>3=completely<br>4=no opinion	5
translated	The protocols you have	1=not at all	5

	worked with are : well translated (only for not english-speaking countries)	2=partially<br>3=completely<br>4=no opinion	
detailed	The protocols you have worked with are : detailed	1=not at all<br>2=more or less<br>3=sufficiently<br>4=too much<br>5=no opinion	6
long	The protocols you have worked with are : long	1=not at all<br>2=more or less<br>3=sufficiently<br>4=too much<br>5=no opinion	6
amend	How many amendements do you have in average per protocol?	1=0<br>2=1 - 2<br>3=3 - 5<br>4=> 5<br>5=I do not know<br>6=Other	8
ic	How well are the study procedures described in the protocol adapted to your specific setting? Informed consent procedure including documentation	1-5<br>1=poorly adapted<br>5=well adapted<br>6=no opinion	9
criteria	How well are the study procedures described in the protocol adapted to your specific setting? Inclusion and exclusion criteria	1-5<br>1=poorly adapted<br>5=well adapted<br>6=no opinion	9
incentiv	How well are the study procedures described in the protocol adapted to your specific setting? Participants incentives to participate in the trial	1-5<br>1=poorly adapted<br>5=well adapted<br>6=no opinion	9
recruit	How well are the study procedures described in the protocol adapted to your specific setting? Recruitment procedure	1-5<br>1=poorly adapted<br>5=well adapted<br>6=no opinion	9
data	How well are the study procedures described in the protocol adapted to your specific setting? Data and information to be collected	1-5<br>1=poorly adapted<br>5=well adapted<br>6=no opinion	9
intervent	How well are the study procedures described in the protocol adapted to your specific setting? Medical interventions (e.g. ECG)	1-5<br>1=poorly adapted<br>5=well adapted<br>6=no opinion	9

procedure	How well are the study procedures described in the protocol adapted to your specific setting? Medical procedures and decisions (e.g. administration of drugs, treatment of concomitant diseases and emergencies)	1-5<br>1=poorly adapted<br>5=well adapted<br>6=no opinion	9
reporting	How well are the study procedures described in the protocol adapted to your specific setting? Safety reporting and management	1-5<br>1=poorly adapted<br>5=well adapted<br>6=no opinion	9
follow	How well are the study procedures described in the protocol adapted to your specific setting? Follow-up procedure	1-5<br>1=poorly adapted<br>5=well adapted<br>6=no opinion	9
workforce	How well are the protocols adapted to…? Amount of workforce available	1-5<br>1=poorly adapted<br>5=well adapted<br>6=no opinion	10
infra	How well are the protocols adapted to…? Infrastructure available	1-5<br>1=poorly adapted<br>5=well adapted<br>6=no opinion	10
particip	How well are the protocols adapted to…? Availability and needs of trial participants	1-5<br>1=poorly adapted<br>5=well adapted<br>6=no opinion	10
practice	How well are the protocols adapted to…? Daily clinical practice	1-5<br>1=poorly adapted<br>5=well adapted<br>6=no opinion	10
ethic	How well are the protocols adapted to…? Ethics Committe system	1-5<br>1=poorly adapted<br>5=well adapted<br>6=no opinion	10
authority	How well are the protocols adapted to…? Drug Regulatory Authority system	1-5<br>1=poorly adapted<br>5=well adapted<br>6=no opinion	10
involvp	Are you involved in the study planning of the clinical trials you are working in? (Please choose all that apply)	1=Stimulating the topic as an expert<br>2=Major involvement in protocol writing<br>3=Minor involvement in protocol writing<br>4=Reviewing the protocol<br>5=Participating in prediscussion of protocol<br>6=As a sponsor-investigator	12

		7=Not involved<br>8=Other	
involvw	In which role would your involvement be most helpful within the study planning of the clinical trials you are working in? (Please choose all that apply)	1=Stimulating the topic as an expert<br>2=Major involvement in protocol writing<br>3=Minor involvement in protocol writing<br>4=Reviewing the protocol<br>5=Participating in prediscussion of protocol<br>6=Not involved<br>7=Other	13
opens	Have you ever heard about open source protocol development?	1=Yes, I have heard about it<br>2=Yes, I have heard about it and was participating in an open source protocol development<br>3=No, I have never heard of it<br>4=Other:	14
top	Please tick the top three options you think help or would help to increase the suitability of trial protocols? (Please tick three options)	1=Sponsor to solicit feedback from site on what went wrong in previous trials<br>2=More careful assessment of local context, capacity and culture by sponsor<br>3=Include participant perspective in study planning<br>4=Involvement of local staff in the study planning/ protocol development<br>5=Use open source protocol developement technique<br>6=Single center trials: Adapt the protocol to site and health care specific systems<br>7=Multi center trials: Having committees which consist of investigators from all involved research centres<br>8=Making sure that everybody understands the protocol and knows his role and responsibility in the trial<br>9=Having a kick-off meeting before the study start where issues can be discussed and detected<br>10=Having a dry run before the enrolment of the first patient<br>11=Having a checklist for all the practical steps of the trial	15
country	In which country do you work most of the time?	Open field	17
insti	In what kind of institution are you working in?	1=Clinical research centre<br>2=Hospital<br>3=Field site	18

		4=Other:	
work	What percentage of your working time is spent for work on clinical trials?	1=0 - 25%<br>2=26 - 50%<br>3=51 - 75%<br>4=76 - 100%<br>5=Other	19
dryrun	For which percentage of clinical trials have you had a dry run (definition: a practice of the trial activities with dummy participants before the enrolment of the first participant)?	1=0 %<br>2=25 %<br>3=50 %<br>4=75 %<br>5=100 %<br>6=Other	20
kickoff	For which percentage of clinical trials have you had a kick off meeting where issues were detected and discussed before the start of the study?	1=0 %<br>2=25 %<br>3=50 %<br>4=75 %<br>5=100 %<br>6=Other	21
lesson	For which percentage of clinical trials have you had a lessons learnt meeting after the trial has ended?	1=0 %<br>2=25 %<br>3=50 %<br>4=75 %<br>5=100 %<br>6=Other	22
sponsor	Who was the sponsor of your study?	1=Mostly pharmaceutical companies<br>2=Mostly other than pharmaceutical companies<br>3=Mixed<br>4=I do not know<br>5=Other	23
multi	What percentage of your trials are multicenter trials?	1=0 %<br>2=25 %<br>3=50 %<br>4=75 %<br>5=100 %<br>6=Other	24
